# MDT-HMTL: A Multidimensional Temporal Hierarchical Multi-Task Learning Framework for Myocardial Infarction Detection and State Classification Using PPG Signals

**DOI:** 10.3390/s26196259

**Published:** 2026-10-02

**Authors:** Hui Hui, Jun Wang, Jinxiu Cheng, Huimeng Zhao, Zhong Ji

**Affiliations:** 1 College of Bioengineering, Chongqing University, Chongqing 400030, China; 202319021079t@stu.cqu.edu.cn (H.H.); 202319131113@stu.cqu.edu.cn (J.W.); chengjinxiu@stu.cqu.edu.cn (J.C.); zhaohuimeng@stu.cqu.edu.cn (H.Z.); 2 Key Laboratory of Biorheological Science and Technology, Chongqing University, Ministry of Education, Chongqing 400044, China

**Keywords:** photoplethysmography (PPG), derivative signals, adaptive feature extraction, machine learning, myocardial infarction, wearable health monitoring

## Abstract

**Highlights:**

**What are the main findings?**

- Fuses multi-order PPG signals (PPG, VPG, and APG) with hierarchical temporal modeling for joint MI detection and multi-stage clinical classification.
- Dynamically captures continuous disease-state transitions from multi-day PPG recordings, enabling non-invasive tracking of MI progression.

**What are the implications of the main findings?**

- Hierarchical temporal modeling extends PPG analysis from static screening to longitudinal assessment of MI progression and recovery.
- Offers a practical foundation for non-invasive risk tracking and decision support in wearable IoMT and digital health systems.

**Abstract:**

Continuous assessment of myocardial infarction (MI) using photoplethysmography (PPG) signals has potential for out-of-hospital cardiovascular monitoring, yet conventional single-channel and short-window approaches have limitations in capturing complex hemodynamic variations and dynamic disease-state transitions. To address these limitations, we propose MDT-HMTL, a Multidimensional Temporal Hierarchical Multi-Task Learning framework tailored for simultaneous MI detection and disease-state classification. By integrating multi-channel physiological representations with hierarchical temporal modeling, MDT-HMTL captures local morphological characteristics and longer-range temporal dependencies within a unified multi-task architecture. Evaluation on the MIMIC-III clinical database achieved an average area under the ROC curve (AUC) of 0.908 and an overall accuracy of 86.4%. Furthermore, ablation studies demonstrated the contributions of multi-dimensional representation learning and hierarchical temporal modeling to the overall performance. The proposed framework demonstrates the feasibility of PPG-based MI detection and longitudinal disease-state assessment, highlighting its potential for future non-invasive and wearable-compatible cardiovascular monitoring.

## 1. Introduction

Cardiovascular disease (CVD) remains a leading cause of global mortality and healthcare burden, with Myocardial Infarction (MI) representing a critical, life-threatening acute ischemic condition [1,2]. Continuous monitoring of cardiovascular function and peripheral vascular dynamics is important for early risk identification, dynamic assessment, and long-term disease management [3]. Although standard 12-lead electrocardiography (ECG) and cardiac biomarkers remain central to clinical diagnosis, they are primarily suited to episodic assessment in clinical settings and do not readily support continuous monitoring of peripheral hemodynamic changes [4,5]. Furthermore, multi-lead configurations and sustained skin-electrode contact may reduce comfort and adherence during prolonged ambulatory monitoring [1,6].

With advances in non-invasive sensing technology, peripheral biosignals have gained increasing attention for out-of-hospital cardiovascular monitoring [7,8]. Wearable ECG, photoplethysmography (PPG), and other optical sensing modalities, together with artificial intelligence (AI)-based signal analysis, have created new opportunities for real-time cardiovascular assessment and personalized health monitoring [9,10]. Among these modalities, PPG is particularly attractive because of its low cost, unobtrusive measurement characteristics, and widespread integration into consumer-grade wearable devices [11,12].

PPG-based cardiovascular analysis has progressively evolved from conventional handcrafted descriptors toward richer waveform representations and learning-based approaches. Early investigations focused on spectral characteristics, interbeat variability, and heart rate variability (HRV) for cardiovascular and MI screening [13,14,15,16]. The use of derivative waveforms, including velocity plethysmography (VPG) and acceleration plethysmography (APG), subsequently provided additional information on waveform morphology and vascular dynamics [17,18]. In particular, first- and second-order derivative features have demonstrated their utility in identifying MI-related waveform changes and supporting automated MI screening [19], while complementary waveform-analysis strategies have enabled the extraction of cardiovascular biomarkers from PPG signals [20]. From a physiological perspective, raw PPG and its derivatives constitute a differential hierarchy containing complementary information on blood-volume dynamics and waveform morphology [21,22]. In parallel, PPG-based cardiovascular assessment has increasingly been integrated into Internet of Medical Things (IoMT) platforms and wearable-oriented systems through wireless signal acquisition, smartphone-based analysis, and computationally efficient feature extraction [23,24,25].

More recently, PPG and other optical cardiac sensing approaches have expanded beyond conventional binary disease screening toward fine-grained characterization of cardiovascular and physiological states. Multimodal ECG–PPG–clinical feature integration has enabled discrimination of STEMI, NSTEMI, and old MI, while PPG-based models have demonstrated multi-class classification across diverse cardiovascular conditions [26,27]. Meanwhile, recent photonic cardiac sensing systems combined with machine learning have demonstrated subject-independent decoding of emotional information from cardiac signals, providing complementary evidence that optical cardiac sensing can support cross-subject characterization of physiological states [28]. Together, these advances indicate that wearable and photonic cardiac sensing can support not only disease-oriented screening but also more comprehensive and individualized monitoring of dynamic physiological states.

Despite this progress, several methodological challenges remain. First, multi-order PPG information has not been fully exploited. Most existing approaches either analyze raw PPG and its derivatives separately or rely on handcrafted descriptors [14,16], leaving the physiological coupling among PPG, VPG, and APG insufficiently modeled as a unified representation. Second, temporal modeling remains limited. Conventional PPG-based approaches predominantly operate on short signal segments or aggregated features, which are suitable for point-in-time classification but provide limited capacity to characterize temporal dependencies extending from individual pulse morphology to intra-day fluctuations and cross-day physiological patterns. Third, disease-state characterization remains largely static. Although multi-class cardiovascular classification is becoming increasingly feasible, most existing frameworks assign a fixed disease label to an input segment rather than explicitly modeling relationships among clinically meaningful MI states over time. Consequently, the longitudinal characteristics of MI-related physiological changes remain insufficiently represented in existing PPG-based frameworks.

To address these limitations, we propose MDT-HMTL, a Multidimensional Temporal Hierarchical Multi-Task Learning framework for continuous MI detection and granular state classification using multi-order PPG signals. The framework integrates multi-order differential PPG representations with hierarchical temporal modeling and stage-aware multi-task learning to characterize MI-related cardiovascular states across multiple temporal scales. The main contributions of this study are as follows:

- Multidimensional Cardiovascular Representation: We construct a three-channel differential hierarchy comprising raw PPG, VPG, and APG. This multi-tier representation explicitly extracts subtle vascular dynamics and compensates for the limited representational capability of conventional single-channel analysis.
- Multi-Scale Temporal Architecture: We design a three-stage hierarchical network to model cardiovascular dynamics across multiple temporal scales. By integrating local pulse-wave morphology, intra-day physiological variations, and multi-day longitudinal patterns, the network captures both transient fluctuations and longer-term changes associated with different clinical states of MI.
- Stage-Aware Multi-Task Learning: We implement a joint multi-task learning strategy that simultaneously performs MI detection and clinical stage classification. By incorporating clinically defined MI states as an auxiliary supervision signal, the framework encourages more structured representation learning and provides an additional constraint for model optimization under limited patient-level data.

The main methodological novelty of this study lies in a unified framework that synergizes multi-tier differential PPG representations, multi-scale temporal modeling, and stage-aware multi-task learning. Rather than relying solely on point-in-time waveform classification, the proposed approach links raw optical sensing with longitudinal and clinically informed characterization of MI-related cardiovascular states, providing a potential framework for continuous and interpretable cardiac monitoring.

## 2. Materials and Methods

### 2.1. Datasets

The primary physiological signals and matched clinical metadata were extracted from the Matched Subset of the Medical Information Mart for Intensive Care III (MIMIC-III) Waveform Database [29]. This database contains high-resolution continuous waveforms—including PPG, ECG, and arterial blood pressure (ABP)—recorded at 125 Hz, along with clinical records such as patient demographics, laboratory biomarkers, and International Classification of Diseases, Ninth Revision (ICD-9) codes.

To evaluate the MDT-HMTL framework for continuous MI monitoring, a rigorous screening protocol was applied to select subjects based on two inclusion criteria:

- MI Group: diagnosed with acute myocardial infarction (AMI, ICD-9 code: 410.x) who had continuous, high-quality PPG recordings spanning from ICU admission to clinical stabilization.
- Control Group: Non-cardiovascular disease (non-CVD) patients admitted for non-cardiac conditions (e.g., trauma or elective non-cardiac surgery) with no documented cardiac history.

The finalized cohort consisted of 18 MI patients (mean age: 69 ± 13 years) and 20 non-CVD control subjects (mean age: 54 ± 19 years). The raw PPG recordings were collected over a period of 2–4 days per participant. After removing segments contaminated by poor sensor contact, signal dropout, and low-perfusion-induced signal distortion, the average cumulative duration of usable, quality-controlled PPG signals reached approximately 14 h per subject.

### 2.2. Framework Overview

**Figure 1:**
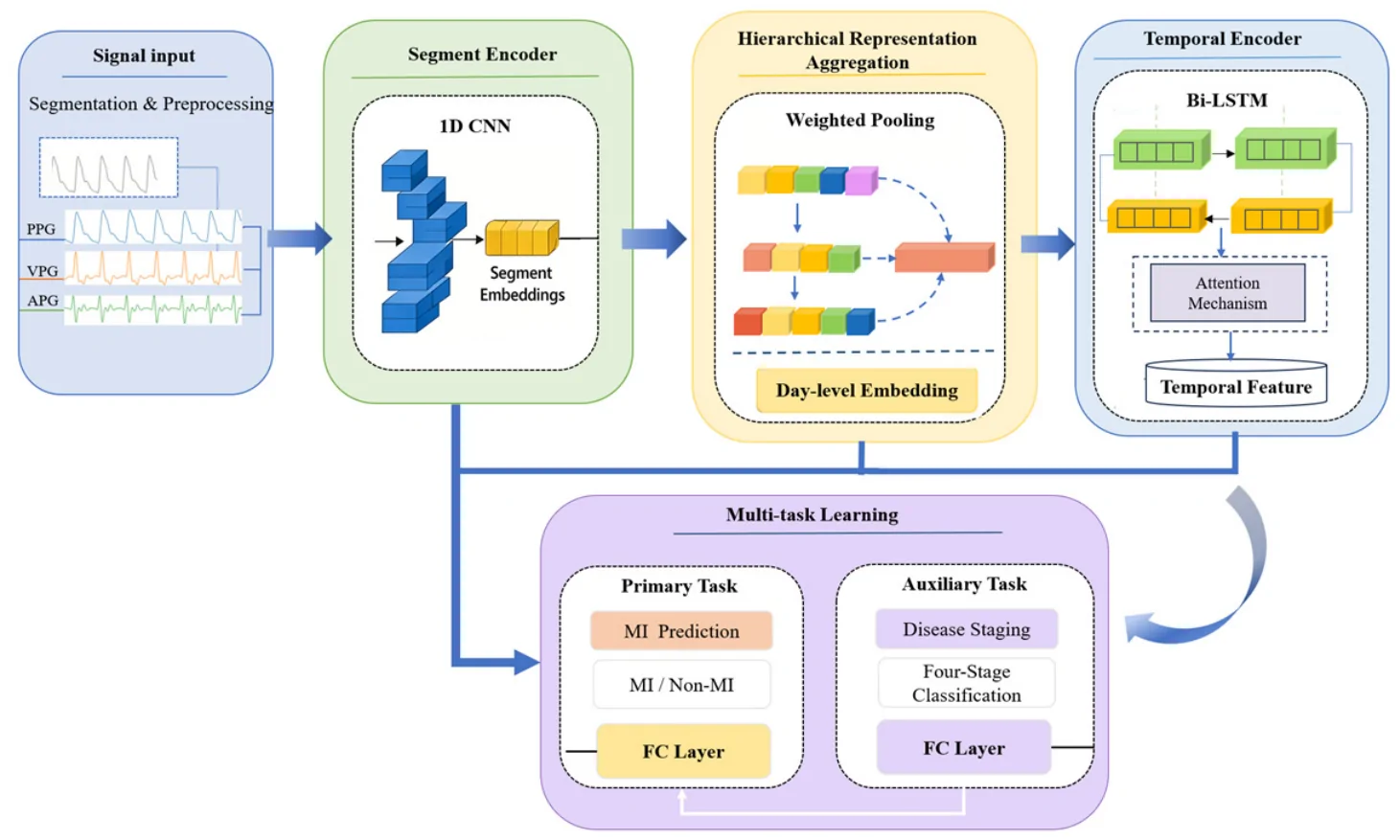
Schematic overview of the proposed MDT-HMTL framework.

The overall workflow of the proposed MDT-HMTL framework is schematically depicted in Figure 1. The methodology integrates four sequential processing modules into a unified pipeline: segment-level feature encoding from synchronized multi-derivative PPG inputs, day-level adaptive aggregation of segment embeddings, longitudinal temporal modeling of long-range disease trajectories, and stage-aware multi-task prediction.

Specifically, short-term pulse wave morphology is first captured across raw and derivative signals, then dynamically aggregated into robust daily representations, and finally decoded across continuous monitoring periods to capture long-term pathological evolution. The entire framework is trained end-to-end under a joint multi-task loss function, enabling the co-optimization of static disease signatures and dynamic trajectory trends for concurrent MI detection and clinical stage classification. The entire framework is optimized end-to-end under a unified multi-task loss to capture both static disease signatures and dynamic trajectory trends.

#### 2.2.1. Signal Preprocessing

PPG signals acquired in continuous clinical environments are inherently vulnerable to motion artifacts, baseline drift, and sensor contact instability [30]. To ensure signal fidelity, we implemented a three-stage preprocessing pipeline consisting of window segmentation and filtering, signal quality assessment (SQA), and stratified temporal sampling.

(1) Window Segmentation and Filtering

Continuous physiological recordings were partitioned into non-overlapping 3-s temporal windows, each containing multiple cardiac cycles for morphological and temporal analysis. Across typical resting and pathological heart rates (40–180 bpm), a 3-s window encompasses approximately 2 to 9 cardiac cycles, with 3–5 beats under typical resting conditions. This provides sufficient cardiac cycles to preserve relevant morphological and temporal waveform characteristics without requiring potentially unreliable beat-level segmentation during arrhythmic events. Each segment was then processed using a 4th-order Butterworth bandpass filter with a passband of 0.5–12 Hz [31]. This frequency range attenuates low-frequency baseline fluctuations and high-frequency noise while preserving the major morphological and harmonic components relevant to derivative generation, including the VPG and APG. Finally, Z-score normalization was applied to each window to reduce amplitude and baseline differences across segments and subjects arising from physiological variability and sensor contact conditions.

(2) Signal Quality Assessment

**Figure 2:**
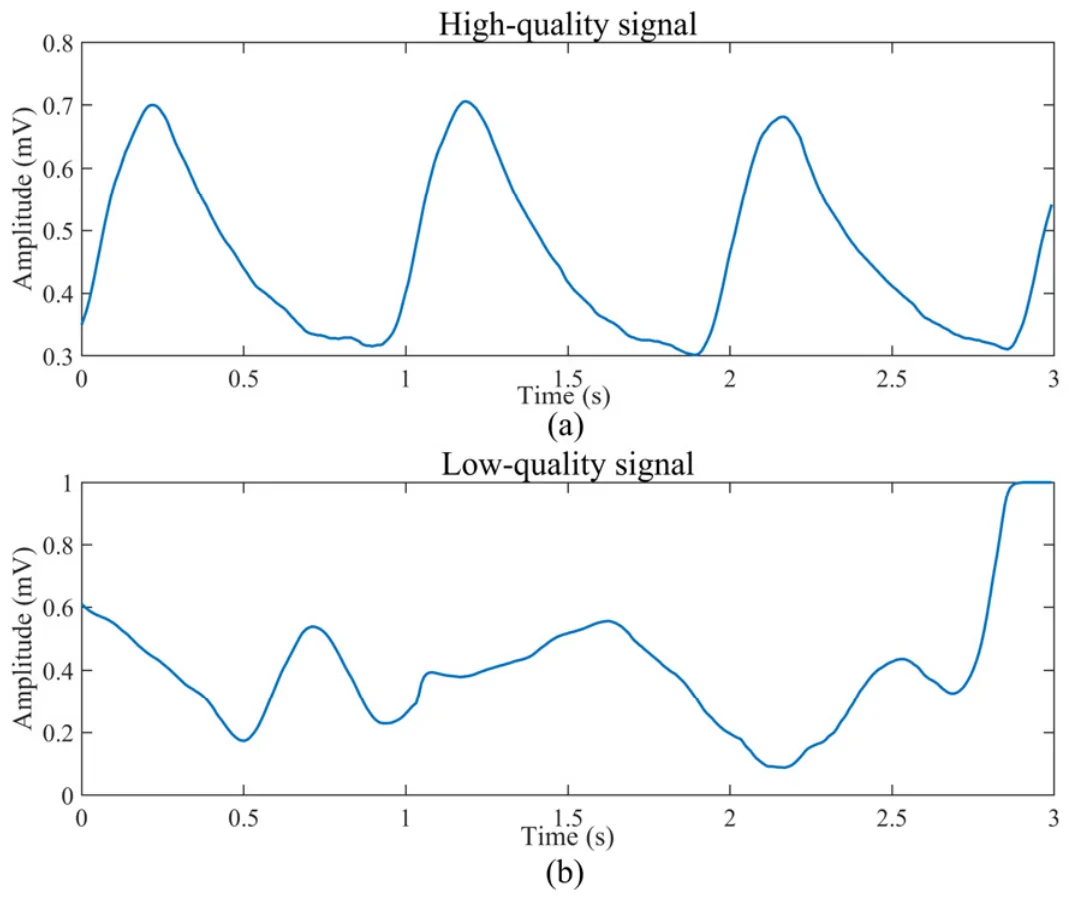
Representative signal quality assessment (SQA) results. (**a**) High-quality PPG signal with complete pulse-wave morphology; (**b**) Low-quality PPG signal corrupted by severe noise and baseline displacement.

To prevent noise propagation stemming from patient movement or temporary sensor detachment, a LightGBM-based SQA model was integrated downstream of filtering [32]. The SQA classifier evaluates waveform integrity by extracting key morphological and statistical descriptors, including skewness, kurtosis, and spectral entropy. Only segments demonstrating complete pulse-wave morphology and high signal-to-noise ratios were retained for downstream modeling, whereas severely distorted windows were automatically discarded (Figure 2). Among all segmented PPG windows, 97.53% of the segments were retained, whereas 2.47% were excluded due to poor signal quality.

(3) Stage-Aware Annotation and Stratified Temporal Sampling

To represent the temporal evolution of MI, we established a clinically informed disease-course annotation strategy based on the clinical framework described in the 2023 ESC Guidelines [33]. Using the initial MI diagnosis date as the temporal anchor, continuously acquired PPG signals were mapped along the longitudinal hospitalization timeline in the MIMIC-III database. Diagnostic records, medication and treatment records, and laboratory test results were also considered to determine the corresponding disease stage. Accordingly, MI recordings were categorized into three sequential disease phases: the Acute Phase (Label 0), Treatment Phase (Label 1), and Recovery Phase (Label 2), while Control recordings were assigned to Label 3. This annotation strategy incorporates disease-course information at the annotation level and provides temporally structured supervision for longitudinal modeling. For the binary MI detection task, Labels 0–2 were mapped to MI, whereas Label 3 was mapped to non-MI. The detailed label definitions and clinical-state criteria are provided in Table 1.

To reduce temporal redundancy in long-term continuous recordings while preserving clinically relevant temporal and stage-wise information, a stratified temporal sampling strategy was applied to the SQA-cleared segment pool. For MI patients, 500 high-quality segments were uniformly sampled from each clinical phase. For non-CVD control subjects, the qualified segments were treated as a single control category because these subjects did not undergo disease-related clinical staging. To address the class imbalance, particularly the different numbers of clinical categories between the MI and control groups, a synergistic dual-level strategy was employed [34]:

- Cohort-Compensated Sampling (CCS): During dataset construction, the number of sampled non-CVD control segments was adjusted relative to the MI segments to maintain an MI-to-control ratio of 1:2. This cohort-level sampling strategy was applied before model training to alleviate the imbalance between the three MI stages and the single control category.
- Cost-Sensitive Learning (CSL): Class-specific penalty weights were incorporated directly into the multi-task loss function during model training to penalize minority-class misclassifications and improve balanced learning across the four stage classes.

Following this preprocessing and sampling pipeline, a refined dataset containing 43,500 high-quality signal segments was obtained for model training, validation, and evaluation.

#### 2.2.2. Multi-Derivative Signal Fusion and Segment-Level Encoder

**Figure 3:**
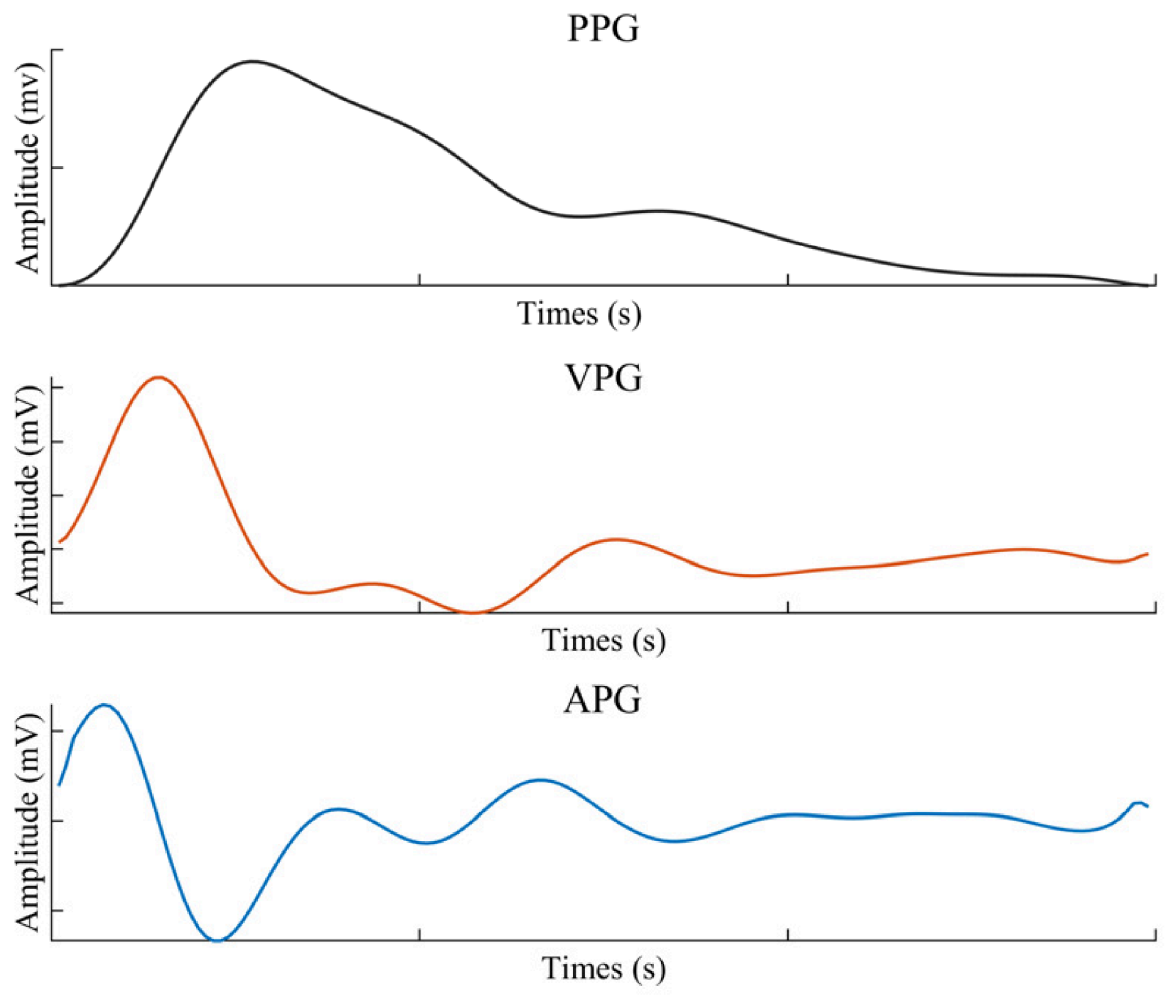
Representative multi-channel physiological inputs comprising synchronized PPG, VPG, and APG signals.

To capture subtle hemodynamic variations in MI, synchronized PPG, VPG, and APG signals are integrated into a multi-channel input tensor $X\in R^{3\times L}$, where L denotes the window length. Channel-wise concatenation merges macro-level pulse morphology from raw PPG with waveform velocity and acceleration from VPG and APG (Figure 3) [35].

**Figure 4:**
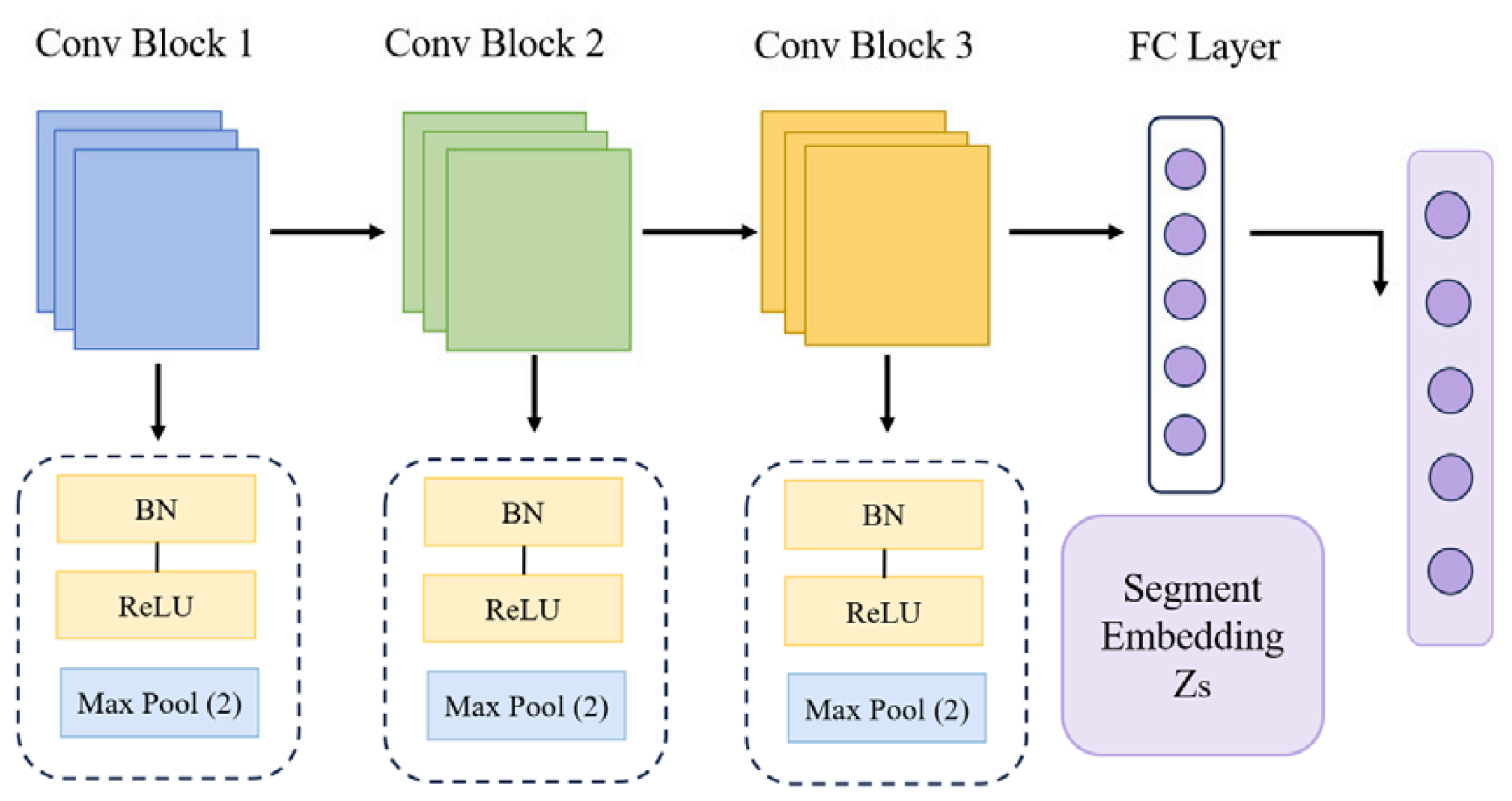
Architectural layout of the segment-level encoder with parameter sharing.

As shown in Figure 4, the multi-channel tensor is processed by a segment-level encoder implemented via a 1D Convolutional Neural Network (1D-CNN) with parameter sharing [36]. The encoder consists of three cascaded convolutional blocks, each incorporating a 1D convolutional layer, Batch Normalization, ReLU activation, and Max-Pooling. To balance global context and localized details, a progressive receptive field scheme is adopted: kernel sizes decrease hierarchically across layers to isolate fine-grained morphology, while the number of feature channels expands to increase representational depth. Finally, a Global Average Pooling (GAP) layer condenses the feature maps into a fixed-dimensional segment embedding $z_{s}\in R^{d}$.

#### 2.2.3. Hierarchical Temporal Aggregation and Progression Modeling

Addressing the complexity of continuous multi-day monitoring, we design a two-stage temporal modeling scheme consisting of intra-day adaptive aggregation and cross-day longitudinal modeling.

(1) Intra-Day Adaptive Aggregation

Intra-day signal collection inherently exhibits physical fluctuations. To mitigate noise, a norm-based adaptive weighting strategy is adopted. Given a set of N segment embeddings ${\left\{z_{s,i}\right\}}_{i=1}^{N}$ extracted from monitoring day d, the representative daily vector $z_{d}$ is calculated as a weighted sum:

(1) $$z_{d}=\sum_{i=1}^{N}\omega_{i}z_{s,i}$$

where the adaptive weight $\omega_{i}$ reflects the feature saliency of each segment, derived from its $l_{2}$ norm and normalized across all segments within day *d* via Softmax:

(2) $$\omega_{i}=\frac{{\left\|z_{s,i}\right\|}^{2}}{\sum_{j=1}^{N}{\left\|z_{s,j}\right\|}^{2}}$$

This norm-based weighting mechanism automatically suppresses low-energy or distorted segments while assigning greater attention to feature-rich pulse waves, producing a stable daily summary representation.

(2) Cross-Day Temporal Dependency and Attention

To track longitudinal disease progression across continuous monitoring days, a two-layer stacked Bidirectional Long Short-Term Memory (BiLSTM) network is employed [37]. For a sequence of daily vectors $\left\{z_{1},z_{2},\ldots ,z_{T}\right\}$ up to day D, the BiLSTM processes the forward and backward dynamics at time step $t\in \left\{1,\ldots ,D\right\}$:

(3) $$h_{t}=\left[\vec{h_{t}},\overset{\leftarrow }{h_{t}}\right]$$

A dropout layer $\left(p=0.3\right)$ is inserted between the two BiLSTM layers to mitigate overfitting.

Considering that the clinical significance of monitoring data varies over time, especially during critical disease transition periods, we introduce a temporal attention mechanism to adaptively aggregate hidden states. Given a learnable query vector q, the attention weights $\alpha_{t}$ and the global sequence representation v are calculated as

(4) $$\alpha_{t}=\frac{exp\left(q^{T}h_{t}\right)}{\sum_{j=1}^{L}exp\left(q^{T}h_{j}\right)}$$

(5) $$v=\sum_{t=1}^{D}\alpha_{t}h_{t}$$

where $h_{t}$ denotes the bidirectional hidden state at day *t*. This dynamic attention mechanism enables the framework to selectively focus on critical pathological transition windows, generating a comprehensive trajectory embedding v for downstream multi-task classification.

#### 2.2.4. Disease-Aware Hierarchical Multi-Task Learning

**Figure 5:**
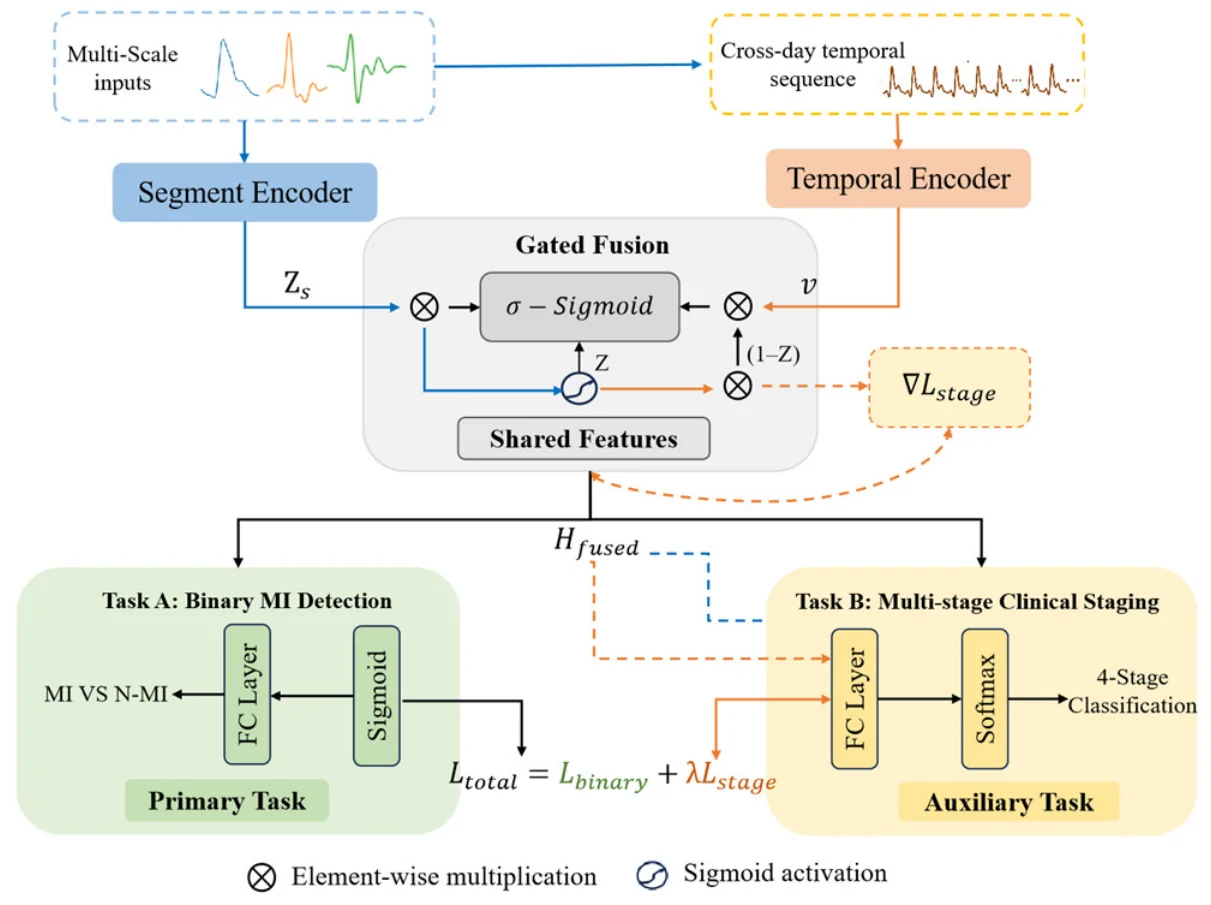
Architectural schematic of the disease-aware hierarchical multi-task learning framework. Notation: Solid arrows: main feature flow. Blue dashed arrows: fine-grained supervised segment-level features for disease stage-wise differences; orange dashed arrows: temporal features.

To simultaneously capture transient hemodynamic abnormalities and longitudinal disease evolution, a disease-aware hierarchical multi-task learning framework is introduced, as schematically depicted in Figure 5. The framework dynamically integrates segment-level and sequence-level representations and jointly optimizes primary MI detection alongside auxiliary clinical stage prediction.

(1) Adaptive Gated Feature Fusion

MI exhibits heterogeneous clinical manifestations across its progression phases—ranging from acute pulse wave distortions to gradual physiological stabilization. Consequently, the relative importance of local morphological signatures versus long-term temporal trends varies across disease states. To dynamically balance these multi-scale features, an adaptive gated fusion mechanism is employed [38].

Let $z_{s}\in R^{d}$ denote the segment-level representation and $v\in R^{2d_{h}}$ denote the temporal representation. The dynamic fusion gate is calculated as:

(6) $$g=\sigma \left[W_{g}\cdot \left[z_{s};W_{u}v\right]+b_{g}\right]$$

where  σ  denotes the sigmoid function, $\left[\cdot ;\cdot \right]$ represents feature concatenation, $W_{u}\in R^{d\times 2d_{h}}$ maps the sequence vector to the segment feature dimension, and $W_{g}$, $b_{g}$ are learnable weight and bias parameters. The fused representation $z_{f}\in R^{d}$ is obtained by the fusion gate g:

(7) $$z_{f}=g⊙z_{s}+\left(1-g\right)⊙W_{u}v$$

where ⊙ denotes element-wise multiplication. When obvious waveform abnormalities exist, higher weights are assigned to segment-level features, making the model focus on discriminative local morphological features related to acute cardiovascular lesions. Conversely, when local waveform changes are subtle or noise-contaminated, higher weights are assigned to temporal representations to leverage stable longitudinal evidence.

(2) Parallel Task Architecture

To incorporate continuous progression-aware supervision beyond static binary labels, the shared representation $z_{f}\;$ is simultaneously routed into two parallel prediction heads:

- Primary MI Detection Head: A binary classification branch predicting disease occurrence (${\hat{y}}_{det}\in \left[0,1\right]$).
- Auxiliary Stage Classification Head: A four-class classification branch predicting specific clinical phases (${\hat{y}}_{stage}\in R^{4}$).

By sharing the multi-scale fused backbone $z_{f}$, stage-level supervision enforces an implicit regularization constraint, compelling the network to preserve fine-grained temporal progression trajectory details alongside disease-discriminative features.

(3) Joint Multi-Task Loss Function

To address class imbalance and maximize the utility of stage-aware annotations, the framework is optimized end-to-end using a weighted multi-task loss function:

(8) $$L_{total}=L_{binary}+\lambda L_{stage}$$

where $L_{binary}$ and $L_{stage}$ denote the Binary Focal Loss and Categorical Cross-Entropy Loss, respectively. Focal loss focuses optimization on difficult samples with subtle hemodynamic abnormalities, while auxiliary stage classification loss enforces progression-aware regularized constraints. The weighting coefficient λ is set to 0.3 via grid search, ensuring that MI detection remains the primary objective while leveraging stage prediction for complementary supervision.

### 2.3. Model Training

A patient-level five-fold cross-validation scheme was implemented to ensure rigorous subject-independent evaluation and prevent data leakage. Patient ID was used as the grouping unit for cross-validation. Thus, all segment-level windows and day-level representations derived from the same patient were assigned exclusively to one fold, preventing data from the same subject from being shared between the training and validation sets. In each fold, four folds (80% of the subjects) were used for model training, while the remaining fold (20% of the subjects) was held out for validation and performance evaluation. Day-level aggregation was performed within the corresponding patient-specific data, without mixing representations from different patients across folds. Therefore, the prevention of cross-contamination was primarily ensured by the patient-level grouping strategy rather than by the aggregation operation itself. The final performance was reported as the mean performance across the five cross-validation folds.

The proposed MDT-HMTL framework was trained in an end-to-end manner using the Adam optimizer to minimize the imbalance-aware hierarchical joint loss described in Section 2.2.4. Model optimization relied on mini-batch gradient descent paired with a cosine annealing learning rate scheduler to dynamically adjust the learning rate during training. Overfitting was mitigated through an early-stopping mechanism, which automatically terminated training if validation loss failed to improve for eight consecutive epochs. The detailed architectural parameters and hyperparameter configurations are summarized in Table 2.

## 3. Results

This section presents a multi-dimensional experimental validation to evaluate the diagnostic performance, robustness, and clinical stability of the proposed MDT-HMTL framework. The systematic evaluation includes segment-level classification performance, overall discriminative capability at the patient-level, fine-grained clinical stage classification, and comprehensive ablation experiments.

### 3.1. Evaluation Criteria

Model performance was comprehensively evaluated using standard clinical diagnostic metrics: Accuracy (ACC), Precision (Pre), Sensitivity (Sens, or Recall), Specificity (Spec), and F1-score. Additionally, the Area Under the Receiver Operating Characteristic Curve (AUC) was utilized to measure discriminative power, particularly given the class imbalance inherent in physiological data. These metrics are defined as follows in Equations (9)–(13):

(9) $$Accuracy=\frac{TP+TN}{TP+TN+FP+FN}$$

(10) $$Precision=\frac{TP}{TP+FP}$$

(11) $$Sensitivity=\frac{TP}{TP+FN}$$

(12) $$Specificity=\frac{TN}{TN+FP}$$

(13) $$F1_{score}=\frac{2\times Precision\times Sensitivity}{Precision+Sensitivity}$$

where TP, TN, FP, and FN denote the counts of true positives (correctly identified MI cases), true negatives (correctly identified healthy controls), false positives (Type I diagnostic error), and false negatives (Type II diagnostic error), respectively.

### 3.2. Model Performance Evaluation Results

#### 3.2.1. Segment-Level vs. Patient-Level Evaluation

**Figure 6:**
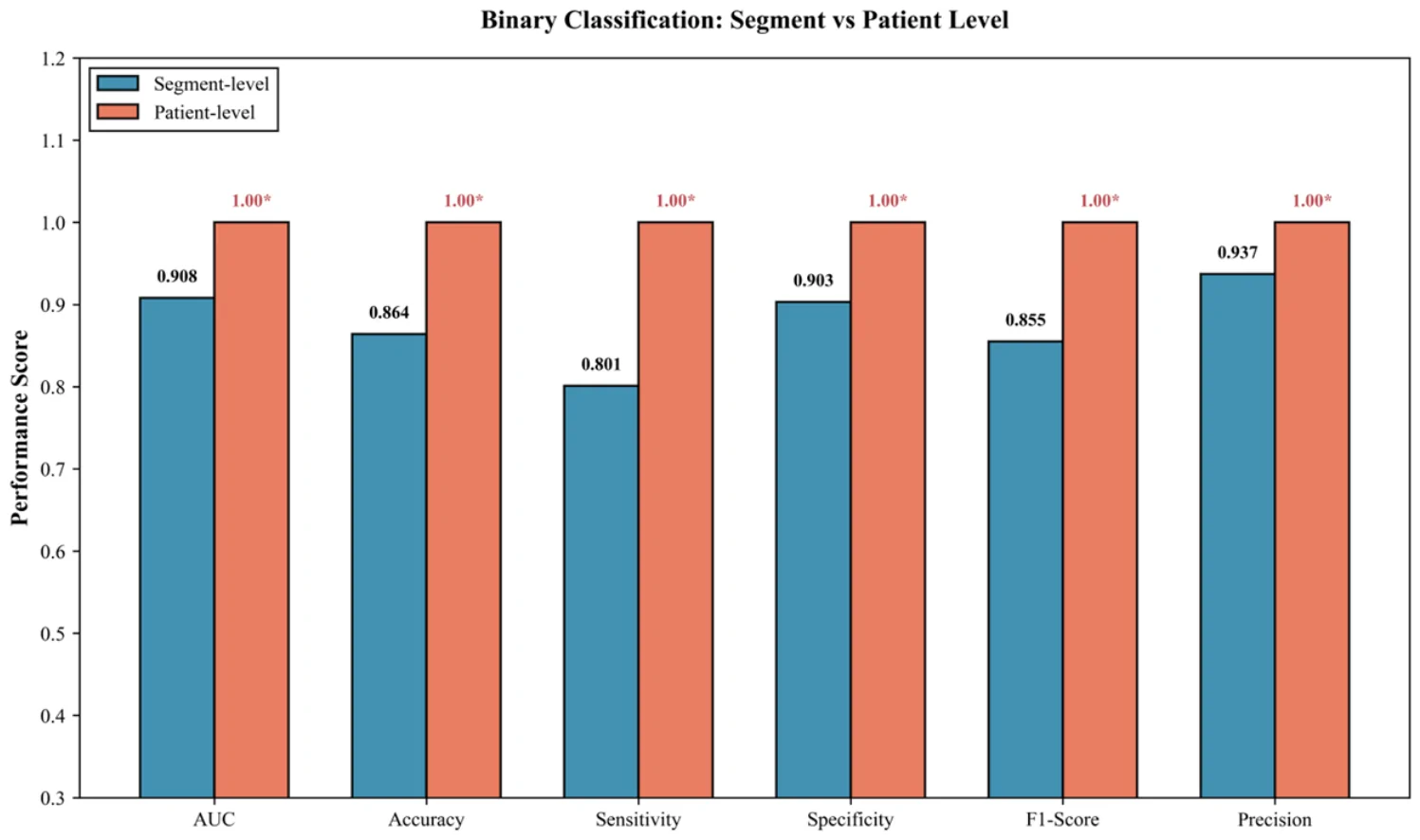
Segment-level and patient-level performance evaluation of the proposed framework for MI identification. The asterisk (*) denotes a perfect score.

The segment-level performance of MDT-HMTL was first evaluated to verify its capacity to capture localized pathological variations. As depicted in Figure 6, the model achieves a segment-level AUC of 0.908, accompanied by a precision of 93.7%, an accuracy of 86.4%, and a sensitivity of 80.1%. Notably, the sensitivity remains lower than specificity, indicating that isolating subtle, transient pathological distortions within individual short-term windows is inherently more challenging than identifying healthy baseline waveforms.

A soft-voting decision strategy was implemented for patient-level prediction to enhance evaluation robustness. Specifically, posterior probabilities across all temporal segments belonging to the same subject were averaged to determine the final subject-level classification. Aggregating multi-segment temporal predictions may help mitigate the influence of changes in body position, transient physiological variations, and random noise, thereby yielding more stable diagnostic decisions than single-segment inference. The patient-level AUC was 1.00 across all five folds. The uncertainty of the pooled patient-level AUC was further estimated using bootstrap resampling, yielding a 95% confidence interval of [1.00, 1.00]. However, patient-level performance may be influenced, to some extent, by the number of available segments per subject and the underlying data distribution. When the number of independent subjects is limited, or the correlation among segments within subjects is relatively high, patient-level aggregation may result in optimistic performance estimates. Further evaluation in larger and more heterogeneous patient populations is warranted to establish the generalizability and clinical applicability of the proposed approach.

#### 3.2.2. Multi-Stage Clinical Phase Stratification

**Figure 7:**
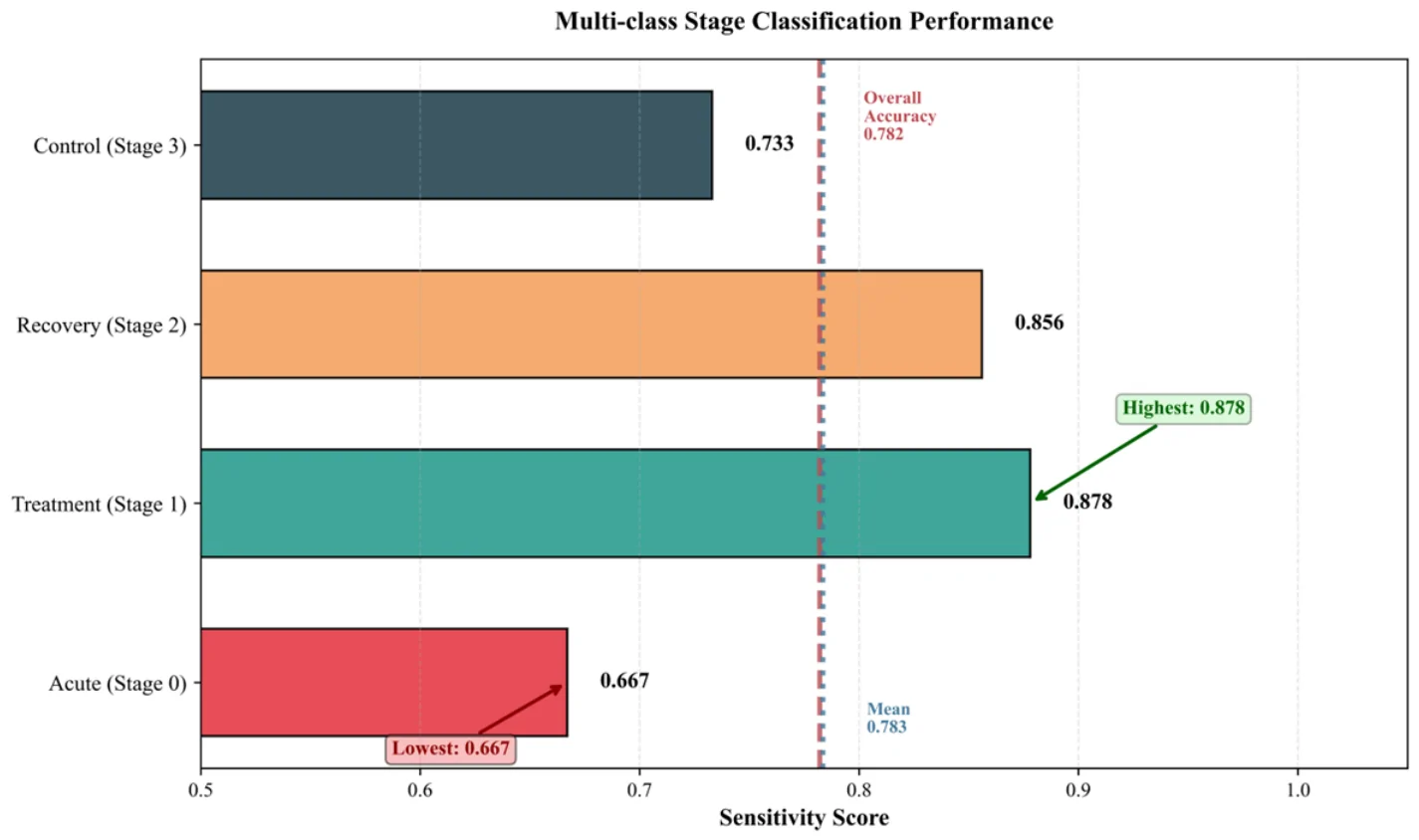
Sensitivity analysis of the model in MI disease stage classification task.

Evaluating the framework’s capacity for continuous disease-stage tracking involved categorizing physiological sequences into four distinct clinical phases: Acute (Stage 0), Treatment (Stage 1), Recovery (Stage 2), and Healthy Control. As summarized in Figure 7, the model achieves a macro-average sensitivity of 78.3% and an overall accuracy of 78.2%.

Among the four evaluation classes, the Treatment and Recovery stages yielded the highest sensitivities (87.8% and 85.6%, respectively). This superior performance is primarily attributed to the stabilized hemodynamic patterns and consistent morphological signatures present during post-treatment hospitalization. Conversely, the Healthy Control group achieved a sensitivity of 73.3%, whereas the Acute stage exhibited the lowest performance. This performance discrepancy is mainly driven by severe hemodynamic volatility and the inherent scarcity of acute-phase continuous recordings.

### 3.3. Ablation Studies

#### 3.3.1. Impact of Multi-Order Derivative Signal Modalities

To evaluate the explicit contributions of multi-order derivative signals in MI identification, we compared four input channel configurations: (a) PPG only, (b) PPG + VPG, (c) PPG + APG, and (d) PPG + VPG + APG (proposed baseline). All variants were trained and evaluated under identical network architectures and patient-level cross-validation splits. The comparative results are summarized in Table 3.

As demonstrated in Table 3, integrating derivative signals yields systematic performance gains, confirming that VPG and APG provide complementary hemodynamic insights beyond raw PPG waveforms. Physiologically, VPG reflects blood flow velocity dynamics and pulse propagation characteristics. By emphasizing the rapid inflection slopes of pulse waves, VPG highlights stable vascular patterns and suppresses false positives, thereby enhancing specificity (93.6%) and precision (92.0%). APG captures acceleration-related vascular characteristics including arterial compliance and wave reflection. These high-order dynamic features exhibit heightened sensitivity to subtle pathological abnormalities caused by MI, enabling the PPG + APG configuration to achieve the highest sensitivity (81.7%).

The superior performance of the full three-channel configuration confirms the synergistic complementarity among these signal modalities. Integrating raw PPG with its first and second derivatives provides a multi-tier physiological representation of cardiovascular hemodynamics, achieving the best overall balance among AUC, accuracy, and F1-score.

#### 3.3.2. Efficacy of Hierarchical Temporal Modeling Modules

To isolate the contribution of the temporal modeling hierarchy, we compared four distinct architectural variations: (a) Case 1, independent segment-level features; (b) Case 2, mean-pooling aggregation; (c) Case 3, learnable weighted aggregation; (d) and Case 4, the proposed MDT-HMTL framework. The patient-level comparison results are summarized in Table 4.

As shown in Table 4, classification performance exhibits progressive gains as temporal modeling depth increases. Lacking temporal continuity, Case 1 yields limited discriminative capability (AUC = 0.733). Uniform mean-pooling in Case 2 improves specificity but causes sensitivity to drop to 0.737, indicating that naive signal averaging dilutes critical, localized pathological signatures. Case 3 introduces adaptive weighting, elevating AUC to 0.797 and sensitivity to 80.3% by dynamically prioritizing feature-rich segments while suppressing noise-corrupted windows. Case 4 achieves ceiling classification performance (AUC = 1.000) under the subject-independent evaluation protocol. This substantial leap confirms the necessity of progressive hierarchical integration—moving seamlessly from segment-level morphology and day-level physiological states to multi-day disease trajectory modeling. This multi-scale strategy effectively mitigates transient signal artifacts while locking onto stable, patient-specific diagnostic markers.

While Case 4 reaches complete separation within the evaluation cohort, this result is primarily facilitated by high temporal sampling density and the multi-segment probability integration mechanism. To prevent overestimating cross-patient generalization, external validation on larger, independent multi-center clinical cohorts remains imperative to confirm broader translational robustness.

#### 3.3.3. Structural Synergy of Multi-Task Learning Architecture

To evaluate the explicit contribution of the auxiliary disease-staging task, we compared the proposed multi-task architecture with a single-task baseline model dedicated solely to MI identification. The segment-level classification results are summarized in Table 5.

As detailed in Table 5, integrating auxiliary disease-stage supervision yields systematic performance gains across overall metrics, achieving an AUC of 0.908, an accuracy of 86.4%, and an F1-score of 85.5%. The most notable enhancements appear in specificity (90.3%) and precision (93.7%), which improve by 2.2% and 2.4%, respectively, over the single-task baseline. This multi-task constraint provides beneficial inductive bias, compelling the shared backbone to extract fine-grained morphological features across distinct clinical phases. By capturing subtle phase-specific wave signatures while filtering out non-diagnostic variations, joint optimization substantially reduces false-positive detections without sacrificing sensitivity.

### 3.4. Model Interpretability and Pathological Alignment

Bridging the semantic gap between deep latent representations and clinical cardiovascular physiology involved a multi-level interpretability analysis incorporating feature space dynamics, long-term risk tracking, and morphological attribution.

#### 3.4.1. Latent Dynamics and Longitudinal Trajectory Tracking

**Figure 8:**
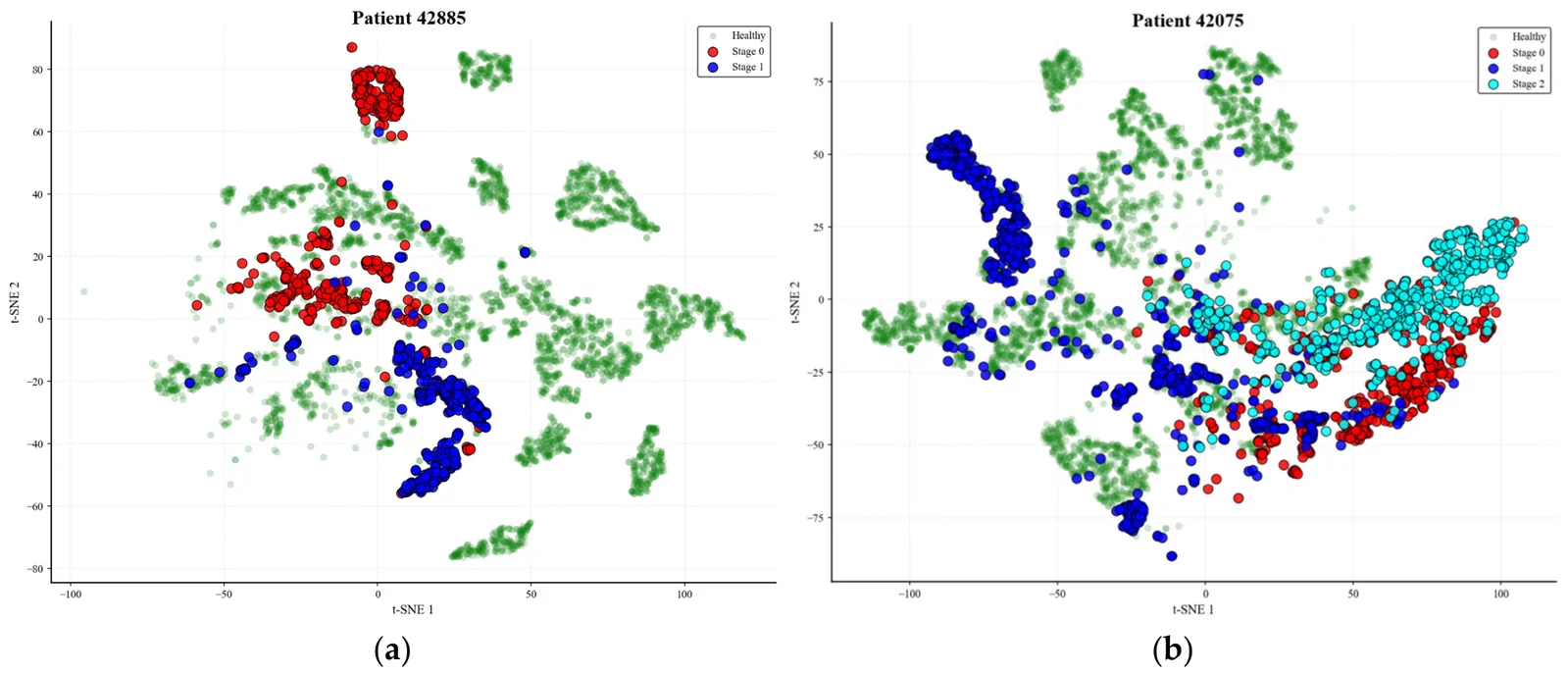
t-SNE visualization of fused feature representations from representative patients. (**a**) Discrete cluster distribution (Patient 42885); (**b**) continuous transitional trajectory (Patient 42075).

Projecting high-dimensional fused feature vectors $z_{f}$ onto a two-dimensional manifold via t-Distributed Stochastic Neighbor Embedding (t-SNE) reveals distinct topological patterns during recovery (Figure 8). For discrete progression modes Figure 8a the Acute and Treatment stages form well-separated clusters, reflecting sudden physiological shifts driven by therapeutic interventions. Conversely, continuous recovery modes (Figure 8b) exhibit a smooth geometric trajectory across disease domains, capturing gradual, non-linear pathological evolutions with highly overlapping transient states, demonstrating that the model recovers true pathological evolution without enforcing artificial categorical boundaries.

These topological distributions provide a mechanistic explanation for the stage-dependent sensitivity variations presented in Figure 7. When physiological transitions between adjacent clinical stages are continuous rather than discrete, classification becomes inherently more challenging. Overall, these findings confirm that MDT-HMTL captures the complex non-linear dynamics of MI recovery beyond the capability of traditional discrete classification paradigms.

**Figure 9:**
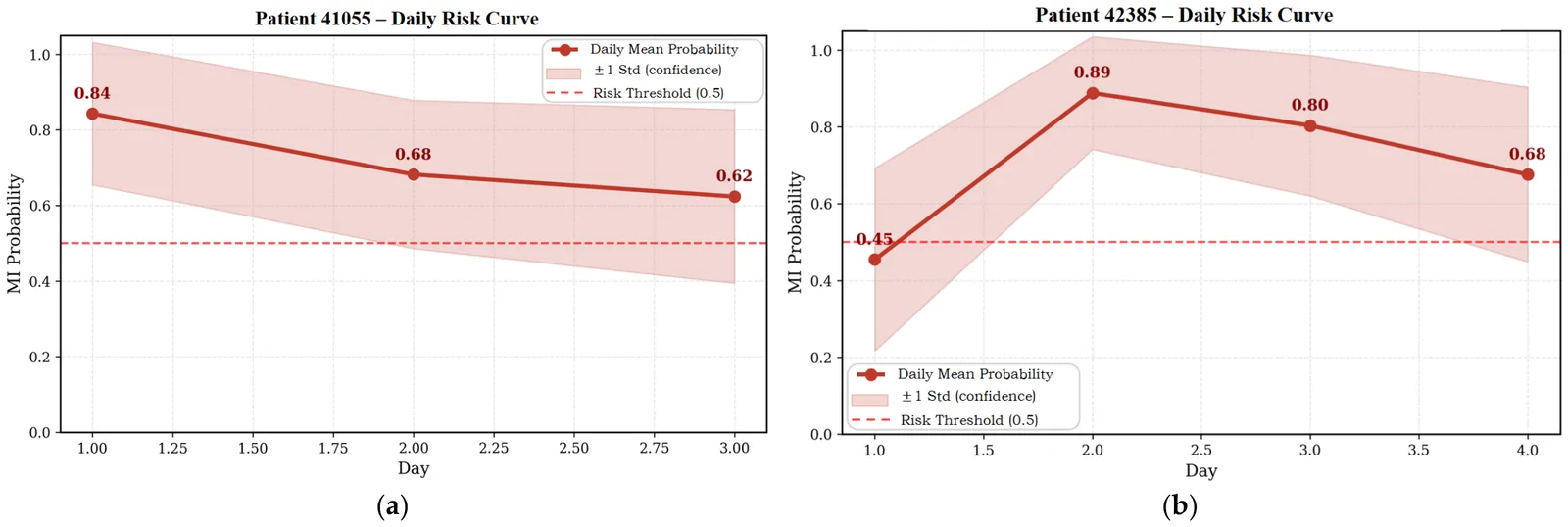
Long-term evolution of MI prediction probabilities under continuous monitoring scenarios. (**a**) Monotonic risk decay (Patient 41055); (**b**) non-linear state transition (Patient 42385).

These continuous topological dynamics directly translate into longitudinal prediction probability trajectories Figure 9. For Patient 41055 (Figure 9a), daily risk monotonically decays from 0.84 to 0.62, mirroring favorable post-intervention recovery. In contrast, Patient 42385 (Figure 9b) displays a non-linear trajectory, with risk surging from 0.45 to 0.89 before stabilization. These trends demonstrate the framework’s capability to track individualized disease progression under continuous monitoring.

#### 3.4.2. Morphological Signal Attribution via Grad-CAM

Gradient-weighted Class Activation Mapping (Grad-CAM) [39] was implemented to visualize discriminative regions of the input waveforms. For target class *c*, the channel weight $\alpha_{k}^{c}$ and the heatmap $L_{Grad-CAM}^{c}$ are defined as

(14) $$\alpha_{k}^{c}=\frac{1}{T}\sum_{t=1}^{T}\frac{\partial y^{c}}{\partial A_{t}^{k}}$$

(15) $$L_{Grad-CAM}^{c}=ReLU\left(\sum_{k}\alpha_{k}^{c}A^{k}\right)$$

where *T* denotes the temporal length, $y^{c}$ represents the class score, and $A_{t}^{k}$ represents the activation of the last convolutional layer.

**Figure 10:**
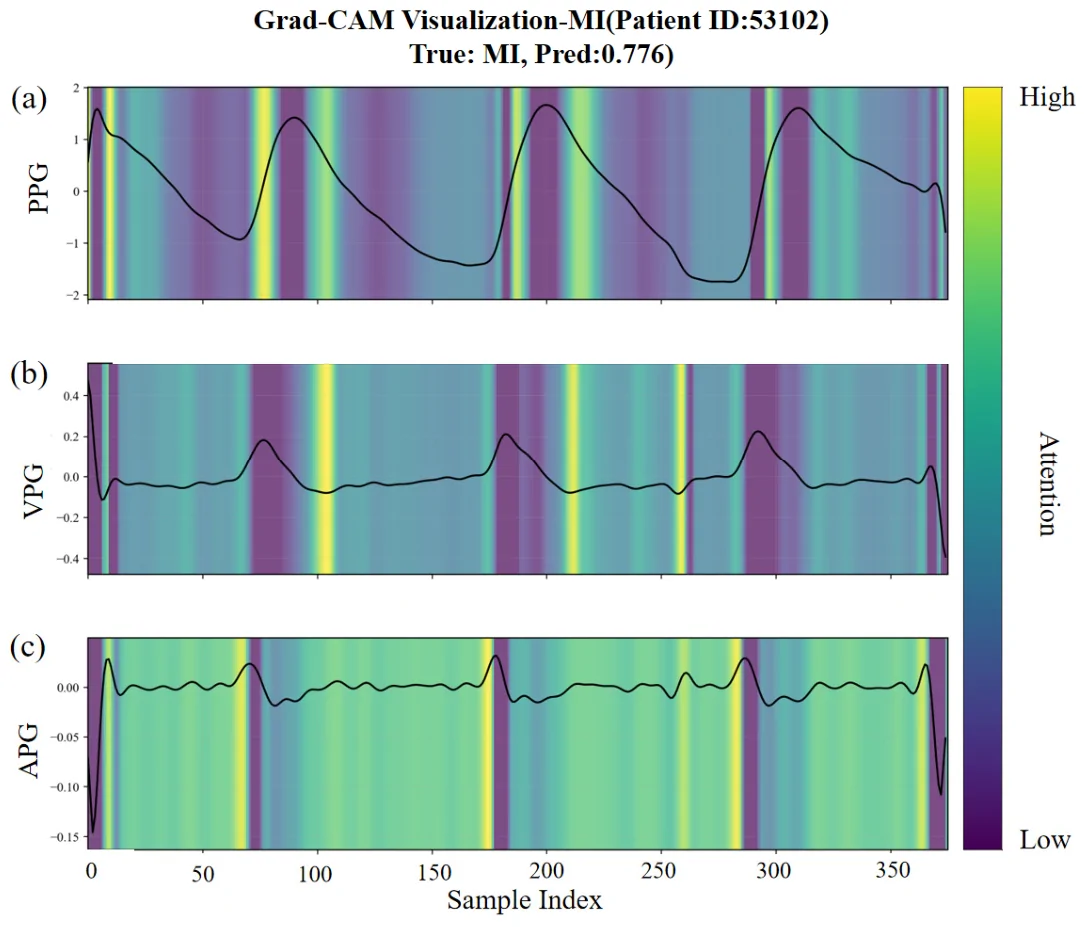
Grad-CAM multi-channel attention visualization for a representative post-MI patient sample, highlighting prominent morphological focus across PPG, VPG and APG channels. (**a**) PPG; (**b**)VPG; (**c**) APG Grad_CAM visualization.

**Figure 11:**
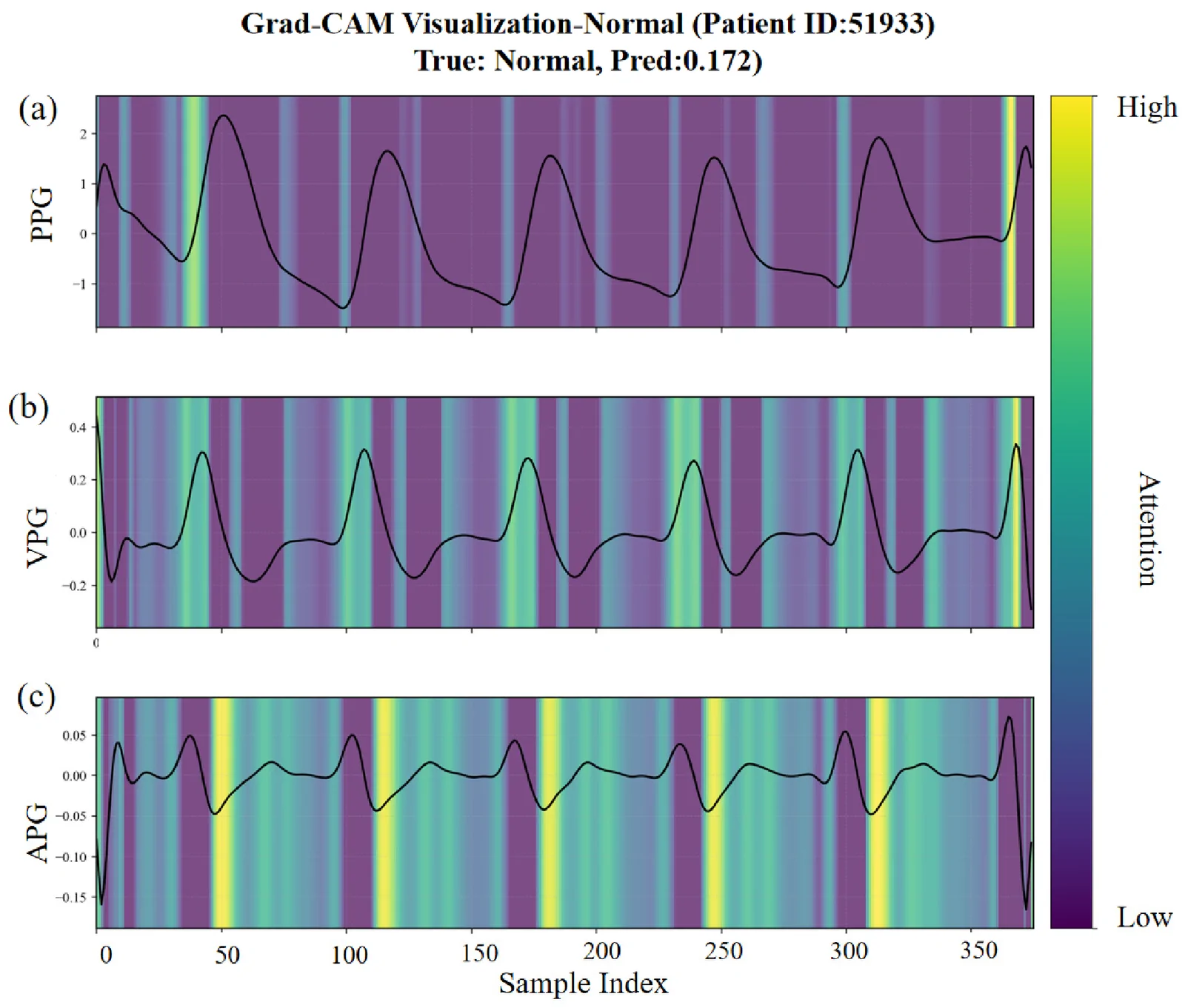
Grad-CAM multi-channel attention visualization for a healthy control sample, illustrating stable and uniform morphological tracking across PPG, VPG, and APG channels. (**a**) PPG; (**b**) VPG; (**c**) APG Grad_CAM visualization.

The generated heatmap is projected onto synchronized PPG, VPG and APG signals to locate the physiological features that determine model decisions (Figure 10 and Figure 11). The black curve denotes the raw physiological waveform, and the background color represents the Grad-CAM attention intensity.

As shown in Figure 10, the model presents channel-specific attention patterns consistent with post-MI hemodynamic changes. The PPG channel mainly focuses on the systolic upstroke and early diastolic decay, reflecting impaired myocardial contractility and reduced peripheral perfusion [40]. The VPG channel highlights the maximum deceleration point in the systolic phase, capturing abnormal ventricular ejection dynamics caused by left ventricular dysfunction [41]. The APG channel focuses on the d- and e-wave regions, indicating enhanced arterial wave reflection and increased arterial stiffness caused by compensatory vasoconstriction [42].

Conversely, healthy control samples (Figure 11) exhibit localized activation restricted to APG regions associated with baseline vascular compliance, with no abnormal focus on the PPG main peak or VPG inflection points. This clear distinction confirms that the framework relies on stable, pathophysiologically meaningful landmarks rather than background noise, ensuring high diagnostic specificity.

### 3.5. Computational Complexity Analysis

High computational efficiency is an important consideration for future wearable cardiovascular monitoring applications [43]. The proposed MDT-HMTL framework contains 325,637 trainable parameters, corresponding to approximately 1.24 MB of model weight storage under FP32 precision. When represented using INT8 precision, the model weight storage is estimated to decrease to approximately 0.33 MB, substantially reducing the memory requirement for model storage [44].

The inference efficiency of MDT-HMTL was evaluated on an Intel Core Ultra 9 285H platform. The average inference latency per sample was 87.506 ± 6.289 ms, corresponding to a throughput of 11.5 ± 0.8 samples/s. Given the 3-s analysis window, the measured inference time is substantially shorter than the corresponding signal acquisition interval, indicating a relatively low computational overhead for continuous inference.

As summarized in Table 6, the compact model size and computational efficiency of MDT-HMTL suggest that the framework may be suitable for further evaluation on resource-constrained edge platforms and wearable-oriented systems [45]. These findings provide preliminary evidence supporting its potential applicability in future wearable cardiovascular monitoring scenarios.

## 4. Discussion

The experimental results suggest that the proposed MDT-HMTL framework facilitates longitudinal MI assessment through the integration of multi-order pulse wave derivatives, multi-scale hierarchical temporal modeling, and fine-grained stage supervision. This design preserves the physical and hemodynamic interpretability of velocity and acceleration plethysmograms (VPG and APG) while leveraging deep temporal architectures to capture longitudinal disease-state transitions that are difficult to reflect using static features alone.

When evaluated alongside existing literature (Table 7), our findings should be interpreted as reflecting a difference in monitoring paradigm rather than a direct quantitative benchmark. Current frameworks [19,23,24] primarily rely on short-duration signal segments or hand-crafted HRV features for point-in-time binary classification. Although these cross-sectional studies report high segment-level accuracy (e.g., >97%), their evaluation settings, including segment-level data partitioning in some studies, may not fully account for intra-subject physiological variability, dynamic changes during recovery, or the need for strict subject-level isolation. Moreover, direct numerical comparisons between MDT-HMTL and existing methods are precluded by substantial heterogeneity in evaluation protocols, patient cohorts, signal modalities, input durations, and clinical task definitions, such as binary screening versus four-class disease-state classification. Within this context, the proposed framework adopts a longitudinal perspective by modeling multi-day physiological trajectories and integrating temporal information across acute, treatment, and recovery phases under patient-independent validation. This perspective complements existing point-in-time classification approaches and provides a basis for investigating PPG-based longitudinal disease-state assessment in patients with MI.

In terms of potential practical applications, MDT-HMTL provides a foundation for trend-oriented decision support in digital health systems. When integrated into wearable Internet of Medical Things (IoMT) platforms, the framework could potentially support post-discharge monitoring, home-based elderly care, and chronic condition management. By continuously modeling individualized risk trajectories and offering quantitative interpretability (e.g., tracking systolic upstroke changes via Grad-CAM), the framework can serve as an auxiliary tool to assist clinicians in identifying early physiological deterioration, helping inform timely secondary examinations (e.g., biomarker assays or standard 12-lead ECGs).

Despite these promising findings, several limitations warrant further investigation. First, the framework was evaluated using ICU cohorts from the MIMIC-III database, which may introduce sampling bias toward older and severely ill populations and limit its direct generalizability to broader ambulatory populations. Differences in age, sex, comorbidities, and disease severity may influence peripheral vascular properties and cardiovascular regulation, potentially affecting PPG characteristics independently of myocardial ischemia [46]. Moreover, ICU-specific interventions, including vasoactive medications, fluid resuscitation, mechanical ventilation, and sedation, can substantially alter peripheral hemodynamics and cardiovascular dynamics [47,48]. Consequently, some signal representations learned by MDT-HMTL may partially reflect ICU-specific physiological or therapeutic states in addition to MI-related changes, potentially resulting in a distribution shift when transitioning to free-living ambulatory settings. Although MDT-HMTL is designed to capture longitudinal patterns, its robustness and generalizability across diverse demographic and clinical settings require further empirical validation.

Second, the use of high-order mathematical derivatives, while beneficial for extracting subtle morphological characteristics from PPG signals, may amplify high-frequency noise and motion artifacts. This limitation may become more pronounced in ambulatory environments with body movement, sensor displacement, and varying contact pressure, highlighting the need to further evaluate the robustness of derivative-based representations under real-world conditions. Future studies will therefore focus on validating MDT-HMTL in larger, multi-center ambulatory cohorts with diverse activity profiles, cardiovascular comorbidities, and medication regimens. Domain adaptation and domain generalization techniques will also be explored to mitigate the ICU-to-ambulatory distribution gap. In addition, hardware-oriented optimization strategies, including mixed-precision quantization and structural network pruning [49], will be investigated to facilitate deployment on resource-constrained ARM Cortex-M microcontrollers for real-time, low-power wearable IoMT applications [50].

## 5. Conclusions

This paper proposes a multidimensional temporal framework for PPG-based MI detection and longitudinal disease-state assessment. The results demonstrate the feasibility of integrating multidimensional physiological representations with temporal disease modeling to characterize evolving cardiovascular abnormalities from PPG signals. The proposed framework also enables temporal disease-state classification across different stages of the MI course, supporting the potential of PPG-based longitudinal assessment as a non-invasive approach for cardiovascular monitoring beyond conventional clinical settings. Given its low computational complexity, the framework may have potential for future integration with wearable sensing systems. However, further validation in larger and more diverse cohorts, under real-world recording conditions and using actual wearable devices, is required to establish its generalizability and practical applicability. Future research will therefore focus on multi-center clinical validation, cross-population generalization, robustness under real-world signal disturbances, and hardware-oriented optimization for potential deployment on wearable and IoT edge devices.

## Figures and Tables

**Table 1:** Annotation Criteria and Sample Distribution for MI Staging.

Physiological Stage	Clinical Definition	Original Label	Final Binary Label
Acute Phase	First 24 h post-diagnosis	0	1
Treatment Phase	Ongoing inpatient clinical treatment	1	1
Recovery Phase	Pre-discharge status with clinical improvement	2	1
Control Group	Non-CVD subjects	3	0

**Table 2:** Network architecture parameters and hyperparameter configurations.

Hyperparameter	Value
Conv Filters	32, 64, 128
Kernel Sizes	7, 5, 3
Bi-LSTM Hidden Units	64
Embedding Dimension	128
Dropout Rate	0.3
Batch Size	64
Learning Rate	1 × 10^−3^ → 1 ×10^−6^
Optimizer	Adam
Early Stopping	8 epochs

**Table 3:** Quantitative ablation performance across varied input channel configurations for MI identification.

Input	AUC	Accuracy	Sensitivity	Specificity	F1	Precision
PPG	0.886	0.836	0.799	0.831	0.833	0.878
PPG+VPG	0.895	0.826	0.746	0.936	0.819	0.920
PPG+APG	0.900	0.831	0.817	0.853	0.832	0.857
PPG+VPG+APG	0.908	0.864	0.801	0.903	0.855	0.937

**Table 4:** Patient-level performance comparison of temporal modeling and aggregation strategies.

Module	AUC	Accuracy	Sensitivity	Specificity	F1	Precision
Case 1	0.733	0.764	0.787	0.780	0.747	0.743
Case 2	0.729	0.789	0.737	0.880	0.765	0.853
Case 3	0.797	0.789	0.803	0.830	0.786	0.850
Case 4	1.00	1.00	1.00	1.00	1.00	1.00

**Table 5:** Performance Comparison of Single-Task and Multi-Task Learning Configurations.

Task Configuration	AUC	Accuracy	Sensitivity	Specificity	F1	Precision
Single-Task	0.904	0.842	0.804	0.881	0.843	0.913
Multi-Task	0.908	0.864	0.801	0.903	0.855	0.937

**Table 6:** Computational Resource and Efficiency Evaluation.

Resource Dimension	MDT-HMTL (FP32)	MDT-HMTL (INT8, Estimated)	Typical MCU/SoC Range
Parameters	0.33 Million	0.33 Million	0.1–5.0 Million
Model Weight Storage	~ 1.30 MB	~ 0.33 MB	0.25–4 MB
RAM	~50–150 KB *	~50–150 KB *	96 KB–3 MB
Inference Latency	87.5 ms (Intel Core Ultra 9, FP32)	20–30 ms (Est.) **	—

* Note: Peak activation memory was estimated from the maximum intermediate activation size during inference and does not include input/output buffers, runtime memory, or other system-level memory overhead. ** The INT8 model weight storage represents an estimate based on 8-bit representation of the model parameters. Embedded inference latency after INT8 quantization was not experimentally evaluated in the present study [36]. The em dash (—) indicates that no typical reference range of inference latency for MCU/SoC platforms is available in the existing literature.

**Table 7:** Performance and Architectural Comparison with the Existing Literature.

References	Year	Signal/Input	Input Scale	Methodology	Result	Clinical Focus
Neha [19]	2023	2nd Deriv. PPG	short segments	Feed-forward ANN	Acc: 98% (public), 94% (self-collected)	Point-in-time MI/UA classification
Shrivastava [23]	2024	4-channel PPG (HR/HRV features)	feature-level	Ensemble ML (XGBoost, RF)	Acc: 97.91% (IoMT), 98.83% (BIOPAC)	Supervised MI classification
Hoang [24]	2025	Smartphone PPG	2 min	Logistic Regression	AUC: 0.832; Se: 87.6%; Sp: 62.4%	App-based AMI screening (HRV-based)
Chakraborty [25]	2026	FDPPG (1st Deriv.)	feature-level	Feature-based ML	Acc: 97.58%; Se: 96.77%; Sp: 98.39%	Feature-based MI identification
Proposed	2026	Multi-dimensional PPG	Longitudinal (multi-day)	MDT-HMTL	Acc: 86.4%, Se: 80.1%, Sp: 93.7%, AUC: 0.908	Longitudinal MI progression continuous state tracking

## Data Availability

Publicly available datasets were analyzed in this study. The raw PPG and physiological waveform data can be accessed from the MIMIC-III Waveform Database Matched Subset v1.0 via PhysioNet at https://doi.org/10.13026/c2294b (accessed on 12 March 2025) (or https://physionet.org/content/mimic3wdb-matched/1.0/) (accessed on 12 March 2025).
